# 

**Published:** 1866-11

**Authors:** 


### CONTENTS FOR NOVEMBER.

ORIGINAL CONTRIBUTIONS.

Rush Medical College—Opening of the Twenty-third Annual Session—Introductory Address by J. W. Freer, M.D ........ p. 491

A Case of Placenta Prævia. By John Reid, M.D........ 502

Clinical Lectures on Diseases of the Eye. By E. L. Holmes, M.D...... 507

Local Anæsthesia. By Henry T. Godfrey, M.D........ 513

HOSPITAL REPORTS.

*County Hospital Surgical Clinic.* Strictures of the Urethra—Perineal Section. By Geo. K. Amerman, M.D ........ 514

PROCEEDINGS OF MEDICAL SOCIETIES.

Chicago Medical Society........ 520

Atomizing Tubes—Concussion followed by Jaundice—Suppression of Urine in Cholera—Typhoid Fever—Report of Committee on the New Anæsthetic Agent, Narcotina—Punctured Wound of Fœtus in Utero—Internal Use of Chloroform.

CORRESPONDENCE........ 523

EDITORIAL........ 523

*Book Notices*—A Guide to the Practical Study of Diseases of the Eye, etc. By James Dixon, F.R.C.S., etc........ 523

St. Luke's Free Hospital........ 524

Mortality in the City of Chicago during the Month of September ........ 526

Mortality for October........ 527

Obituary........ 527

THE DEATH OF DR. BRAINARD........ 529

